# A patient presenting with intact sensory modalities in acute spinal cord ischemia syndrome: a case report

**DOI:** 10.1186/1752-1947-5-31

**Published:** 2011-01-26

**Authors:** Omar Abdel-Mannan, Imran Mahmud

**Affiliations:** 1 St John's College, St Giles, Oxford OX1 3JP, UK; 2 St Catherine's College, Oxford OX1 3UJ, UK

## Abstract

**Introduction:**

Acute spinal cord ischemia syndrome is a rare condition comprising a small fraction of neurovascular accidents, the majority of which occur within the cerebral circulation. The circulation of the spinal cord has several unique features that determine the clinical presentation.

**Case presentation:**

In this case of a 67-year-old Caucasian man who came to our emergency department with sudden-onset, severe right-sided pain and bilateral upper limb weakness, an atypical pattern of sensory deficit was observed. In this case report, we review acute spinal cord ischemia syndrome and consider the pathophysiology, diagnostic measures and prognostic factors associated with patient recovery.

**Conclusion:**

Acute spinal cord ischemia syndrome with atypical patterns of sensory deficit is uncommon. Clinicians must consider acute spinal cord ischemia syndrome when assessing all patients with acute neck pain and focal neurological deficits; atypical presentations can present a diagnostic challenge. Current knowledge of the long-term outcome in patients with spinal cord ischemia is based on only a few small studies, some of which are discussed here.

## Introduction

Spinal cord infarction, or death of a macroscopic region of tissue in the spinal cord, is a rare event. Estimates report spinal cord infarction comprising only 1.2% of all strokes and an overall annual incidence of only 12 per 100,000. The pattern of symptoms can predict the vascular territory affected during the ischemic episode [1]. Acute spinal cord ischemia syndrome (ASCIS) is predictably due primarily to pathology in the anterior spinal artery, its feeders or its branches. First described by Spiller in 1909 [2], thrombosis of the anterior spinal artery is often due to fracture of a cervical vertebra or a cervical hyperextension injury. Vascular disease risk factors are present in 50% of patients, and no clear etiological cause is identified in as many as half of patients [3]. In this case study, the patient's spinal cord infarction is likely to have resulted from atherosclerotic changes in the spinal cord vasculature because of our patient's vascular risk factors.

The spinal circulation comprises two paired posterior spinal arteries running down the dorsum of the cord and a single anterior artery found in the median fissure. Up to eight radicular arteries are established during development and supply the anterior spinal artery, the largest of which is the artery of Adamkiewicz between T9 and T11. Mid-thoracic levels are most vulnerable to ischemia (for example, as a result of thrombosis) as there is only one radicular artery supplying the anterior spinal artery in this region, and sparse anastomoses. Extensive collateral circulation tends to protect the posterior arterial territories from vascular disease.

The hallmarks of acute spinal cord infarction are a sudden, apoplectic onset of severe back or neck pain (50-80% of cases) accompanied by paraparesis or paraplegia [4]. The territory of the anterior spinal artery covers the anterolateral and corticospinal tracts, but not the dorsal columns. The classic presentation for anterior spinal artery ischemia or infarct is sensory deficits in the following pattern: distal to the lesion, pain and temperature are lost bilaterally (owing to the involvement of anterolateral spinothalamic tracts), but light touch, vibration and position sense are preserved (owing to sparing of dorsal columns). Muscle weakness (involvement of the corticospinal tract) and sensory loss occur at the spinal cord segmental levels of infarct. Predictably, the syndrome of symptoms varies with the level of the spinal cord in which the lesion occurs.

The causes of ASCIS include arteritis of intrinsic cord vessels and emboli and/or atheromata. Compression by intervertebral discs or embolization of disc fragments can also cause ischemia (often without a history of trauma). Aortic clamping (during aortic surgery) and dissecting aortic aneurysms are also recognized causes.

On admission, it is essential to evaluate any neurological signs and to carefully document the extent of any neurological deficit. Patients should also be monitored for hemodynamic stability, including blood pressure. Routine investigations should include the following:

1. Full blood count, erythrocyte sedimentation rate (ESR), lipid and/or cholesterol levels, a serological test for syphilis and electrolyte analysis;

2. Leukocytosis, which may suggest an infectious cause such as myelitis;

3. Vascular risk factors should be assessed in all patients, including fasting blood glucose, lipids and/or cholesterol, anti-cardiolipin and/or anti-phospholipid syndromes and other coagulation disorders (including thrombocytosis);

4. A vasculitis and/or arteritis screen, including ESR, antinuclear antibodies and complement levels; and

5. Diabetes mellitus, which is present in about half of patients with an epidural abscess, as well as being a risk factor for vascular pathology.

Imaging studies are performed not only to diagnose ischemic regions of the spinal cord that explain the symptoms, but also to exclude any mass or space-occupying lesions that may be compressing the spinal cord or its vascular supply, either intra- or extra-axial. Magnetic resonance imaging (MRI) is the safest and surest way to identify such a lesion, as well as providing important information regarding the integrity of the spinal cord. On T2-weighted (T2W) MRI scans, high signal intensity in the region of the infarct as well as cord enlargement (on T1-weighted images) are common findings [5]. However, it was reported in one recent study that a mere 45% of patients with acute spinal cord ischemia or infarction demonstrate signal intensity changes on T2-weighted MRI scans [6]. It is important to note that MRI scans can show normal spinal cord signals in the first few hours of clinical evolution, and pathological changes appear only in later studies.

The use of diffusion weighted imaging (DWI) is well established in the brain, but much less so in the spinal cord because of technical difficulties while acquiring images. Recent reports, however, suggest that DWI offers greater sensitivity than T2W images in the diagnosis of acute spinal ischemia [7,8] and that changes may be detectable at earlier stages on DWI scans than on T2W images, presenting DWI MRI as the standard for non-invasive diagnostic imaging in the setting of acute spinal infarction.

## Case Presentation

Our patient, a 67-year-old Caucasian man, presented in the morning to the emergency department complaining of a sudden onset of excruciating right-sided arm and neck pain radiating to his head, followed by two episodes of vomiting. Both arms had reduced power from the shoulder distally. He complained of weakness in his legs, but denied any recent trauma, falls or loss of consciousness. On initial examination, shoulder abduction and adduction were absent, and flexion and extension of the elbow joint 2/5 on the Medical Research Council (MRC) power scale. Power in the lower limbs was normal. All modalities of sensation were intact bilaterally in the upper and lower limbs. No cranial nerve abnormalities were detected.

**Figure 1:**
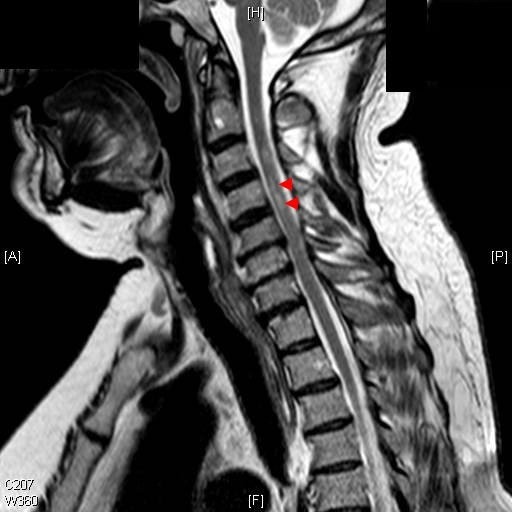
**Axial T2-weighted magnetic resonance imaging (MRI) scan shows abnormally high signal intensity throughout the spinal cord at levels C3-C5**. This is a common finding for regions of infarct.

**Figure 2:**
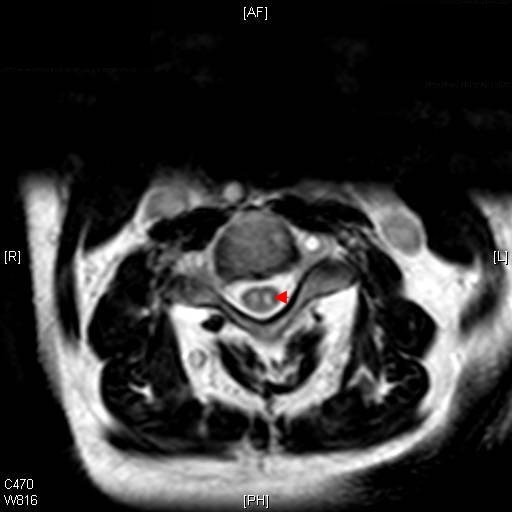
**Sagittal T2-weighted MRI scan showing an abnormally high signal of the spinal cord extending from C3 down to the lower end plate of C5**.

The patient had recently been diagnosed with hypertension and type 2 diabetes. His blood results on admission showed no abnormalities apart from a high glucose level consistent with untreated diabetes. A computed tomography angiogram showed no signs of thrombosis or dissection of the aorta. A spinal MRI scan showed a high-intensity lesion in the anterior two-thirds of the gray matter extending from C3 to C5 (Figures 1 and 2). He was diagnosed with anterior spinal artery ischemia secondary to dissection of a distal vertebral artery. He was started on 300 mg aspirin, and his case was reviewed by both a physiotherapist and a diabetic specialist nurse while he was an in-patient. Within three days of presentation, power in both of his arms had improved (MRC score 4/5), except for abduction of his right shoulder, which he could not lift above the horizontal position.

Spinal cord infarction and ischemia are rare and often difficult to diagnose, but they represent important differential diagnoses of acute spinal symptoms. This case presentation describes an interesting presentation of ASCIS which differs from the classically described symptoms, insofar as the patient had no clinically detectable sensory deficit. This disorder is also associated with significant morbidity and mortality. In this case, the prognosis was favorable. This led us to a review of the literature on the prognosis and outcome of acute spinal cord ischemia.

## Discussion

The standard drug therapy for ASCIS is aspirin, which is based upon the medical recommendation for acute treatment of ischemic stroke of any type. In addition, clopidogrel and a combination of aspirin and controlled-release dipyridamole may be of benefit in reducing the risk of recurrent stroke and death [9]. A number of studies have explored both prognosis and recovery of patients with ASCIS, with the aim of identifying the factors that influence spinal cord infarction outcome (see Table 1). Salvador de la Barrera *et al. *[10] conducted a retrospective study of 36 patients with vascular spinal cord ischemia in which symptom severity was graded according to the American Spinal Injury Association (ASIA)/International Medical Society of Paraplegia (IMSOP) criteria. Assessment of functional outcome was based on wheelchair use or ambulatory ability. The initial severity of symptoms was identified as the strongest predictor of outcome, and this has been confirmed by further studies [11,12]. Those patients lacking voluntary muscle contraction at admission (ASIA groups A and B) were found to be at a higher risk of needing to use wheelchairs. The degree of pathology found on MRI scans (for example, cord enlargement and high-intensity lesions), however, could not be directly correlated to the severity of the neurological syndrome.

**Table 1:** Summary of various prognosis and outcome studies on spinal cord ischemia^a^

Source	Patients	Mean follow-up	Neurological syndrome (%)	Severity of initial motor deficit (%)	Outcome (%)
Salvador de la Barrera *et al. *[10]	36	19.9 ± 30 months	ASAS (100)	ASIA A (19.4)	Full walking ability (18)
				ASIA B (27.8)	Walk with aids (25)
				ASIA C (30.6)	Wheelchair user (57)
				ASIA D (19.4)	Dead (22.2)
Nedeltchev *et al. *[6]	57	4.5 ± 4 years	ASAS (67)	ASIA A (12)	Full walking ability (41)
			PSAS (3)	ASIA B (18)	Walk with aids (30)
			BSS (18)	ASIA C (28)	Wheelchair user (20)
			CSCT (12)	ASIA D (42)	Dead (9)
Cheshire *et al. *[1]	44	1.2 ± 2 years	No data	Paraplegia (57)	Full walking ability (11)
				Paraperesis (41)	Walking with aids (27)
					Wheelchair user (44)
					Dead (18)
Iseli *et al. *[12]	28	6 months	No data	ASIA motor score (mean) 57.22	Full walking ability or ability to walk with aids (25)

**Abbreviations**: ASIA American Spinal Injury Association, ASAS Anterior spinal artery syndrome, PSAS Posterior spinal artery syndrome, BSS Brown-Séquard syndrome, CSCT Complete spinal cord transaction

^a^Adapted with permission from Nedeltchev *et al. *[6]. A comparison of the clinical characteristics and outcome measures reported in four different studies on spinal cord ischemia highlighting the large differences between groups and the scarcity of long-term follow-up studies with large sample sizes.

Advanced age in patients was also identified as a risk factor for unfavorable functional outcome; the mean age of the walking group was 49.2 years compared to 61.4 years in the wheelchair group. For every year older that patients were at presentation, there was a relative risk of 1.14 of remaining wheelchair-bound. Why is age a factor in determining prognosis? One possible explanation for this is that associated co-morbidities and a gradual deterioration in motor learning ability with increased age impede the rehabilitation process, resulting in a poorer outcome.

Most of the current studies fail to address long-term outcome in patients with ASCIS. More recently, Nedeltchev *et al. *[13] conducted a retrospective analysis of 54 patients with a number of ischemic neurological syndromes for a much longer mean follow-up period of 4.5 years to address this question. Clinical improvement and outcome were graded in a similar manner to the study by Salvador de la Barrera *et al. *[10]. Nedeltchev *et al. *confirmed, as previously reported [14], that patients with a severe initial motor deficit of ASIA grade A or B had a poorer outcome. However, age, vascular risk factors and time from symptom onset to maximum severity were not found to be significant predictors of poor prognosis.

## Conclusion

This case report demonstrates an interesting and unique presentation of ASCIS. We observed an unexpected pattern of sensory deficits. Rather than the classical pattern comprising loss of pain and temperature sensation distal to the lesion, with sparing of vibration and position sense, in our patient we detected intact sensory modalities in all dermatomes tested. Our patient had preserved muscle function at the onset of symptoms (with only temporary weakness), which is predictive of a good outcome as shown in the aforementioned prognostic studies. Spinal cord infarction is often serious, leading to paraplegia or even death, and requires prompt diagnosis; our patient was fortunate and suffered only mild disability, and he is currently continuing physiotherapy for further rehabilitation.

## Abbreviations

ASCIS: Acute Spinal Cord Ischemia Syndrome; MRC: Medical Research Council; ASIA: American Spinal Injury Association; IMSOP: International Medical Society of Paraplegia.

## Consent

Written informed consent was obtained from the patient for publication of this case report and accompanying images. A copy of the written consent is available for review by the Editor-in-Chief of this journal.

## Competing interests

The authors declare that they have no competing interests.

## Authors' contributions

OA researched and analyzed the literature for the discussion section, which he wrote. IM carried out the research for the Introduction and wrote that section. Both authors contributed to the case report section equally and were involved in the editing of all the other sections. Both authors read and approved the final manuscript.
